# Oral Antibiotic for Empirical Management of Acute Dentoalveolar Infections—A Systematic Review

**DOI:** 10.3390/antibiotics10030240

**Published:** 2021-02-28

**Authors:** Leanne Teoh, Monique C Cheung, Stuart Dashper, Rodney James, Michael J McCullough

**Affiliations:** 1 Melbourne Dental School, The University of Melbourne, Carlton, Melbourne, VIC 3053, Australia; monique.c.cheung@gmail.com (M.CC.).; stuartgh@unimelb.edu.au (S.D.); m.mccullough@unimelb.edu.au (M.JM.); 2 The National Centre for Antimicrobial Stewardship, Melbourne, VIC 3000, Australia; Rodney.James@mh.org.au

**Keywords:** antibiotics, dental, antibiotic resistance, dentoalveolar, odontogenic

## Abstract

Concerns regarding increasing antibiotic resistance raise the question of the most appropriate oral antibiotic for empirical therapy in dentistry. The aim of this systematic review was to investigate the antibiotic choices and regimens used to manage acute dentoalveolar infections and their clinical outcomes. A systematic review was undertaken across three databases. Two authors independently screened and quality-assessed the included studies and extracted the antibiotic regimens used and the clinical outcomes. Searches identified 2994 studies, and after screening and quality assessment, 8 studies were included. In addition to incision and drainage, the antibiotics used to manage dentoalveolar infections included amoxicillin, amoxicillin/clavulanic acid, cefalexin, clindamycin, erythromycin, metronidazole, moxifloxacin, ornidazole and phenoxymethylpenicillin. Regimens varied in dose, frequency and duration. The vast majority of regimens showed clinical success. One study showed that patients who did not receive any antibiotics had the same clinical outcomes as patients who received broad-spectrum antibiotics. The ideal choice, regimen and spectrum of empirical oral antibiotics as adjunctive management of acute dentoalveolar infections are unclear. Given that all regimens showed clinical success, broad-spectrum antibiotics as first-line empirical therapy are unnecessary. Narrow-spectrum agents appear to be as effective in an otherwise healthy individual. This review highlights the effectiveness of dental treatment to address the source of infection as being the primary factor in the successful management of dentoalveolar abscesses. Furthermore, the role of antibiotics is questioned in primary space odontogenic infections, if drainage can be established.

## 1. Introduction

The majority of dentoalveolar infections arise from necrotic dental pulp, periodontal tissues or pericoronal tissues. An acute dentoalveolar abscess forms from an inflammatory response of the periapical connective tissues, associated with a necrotic pulp. A swelling may develop, in association with resorption of the cortical bone [1]. The key principle for the management of these infections involves local dental treatment by addressing the cause of the infection to establish drainage through the soft tissues, by root canal treatment or by extraction of the offending tooth [2]. Antibiotics are only required as an adjunctive measure when the infection has spread beyond the confines of the tooth and cannot be surgically addressed or shows signs of systemic spread, such as an extra-oral facial swelling, cellulitis or temperature elevation, when the bacterial insult exceeds the capacity of the body’s defence mechanisms [2,3]. In community outpatient dental practice, antibiotics are given empirically, as the standard current practice does not involve pus sampling for microbial investigation [2,4].

Guidelines for the therapeutic use of antibiotics in dentistry differ worldwide [2,5,6,7]. Penicillins are the most frequently prescribed drug class for dental infections, with amoxicillin being most commonly prescribed in most locations worldwide [8,9,10,11]. Dental guidelines in the United Kingdom and United States recommend monotherapy with a penicillin for acute odontogenic infections as first-line treatment [5,6], whereas the recently published Australian guidelines recommend a broader-spectrum combination of a penicillin with metronidazole [2].

The contribution of dental antibiotic prescriptions towards the global public health problem of the development of antimicrobial resistance is an ongoing concern, with dentists being more recently included in antibiotic stewardship initiatives [12,13]. Dental antibiotic prescription accounts for approximately 10% of all antibiotic prescriptions worldwide [14], and it is known from surveys, retrospective studies and prospective audits that overprescribing of dental antibiotics occurs at rates between 55 and 80% [15,16,17,18]. Dental antibiotic prescribing is associated with increased bacterial resistance, especially with regard to the use of metronidazole [19,20], and serious antibiotic-associated adverse events including *Clostridioides* (formerly *Clostridium*) *difficile* infections [21]. Penicillin-resistant odontogenic infections are also associated with increased hospital stays and poorer clinical outcomes [22]. Inappropriately managed dental infections can progress to severe submandibular space infections with associated serious complications, such as sepsis and airway obstruction [23]. Appropriate use and choice of antibiotics in dentistry plays an important role in antibiotic stewardship. 

Odontogenic infections are polymicrobial in nature, consisting of aerobes, facultative anaerobes and aerotolerant, and strict anaerobes [24]. The question of which antibiotic is most appropriate for the management of these infections in a community dental setting is often asked. Many studies have assessed severe odontogenic infections in hospital settings where intravenous antibiotics have been administered and microbial sampling of pus undertaken to determine susceptibility [22,25,26]. However, in a community dental setting, the vast majority of dental infections would be managed with local dental treatment and/or oral antibiotics if the infection has spread beyond the confines of the tooth, but not to the extent that the patient requires hospitalisation. Knowing the best antibiotic for the empirical treatment of dentoalveolar infections where pus sampling is not undertaken is critical to the management of these patients. Therefore, the aim of this study was to investigate the regimens of the oral antibiotics used to manage acute dentoalveolar infections and the subsequent clinical outcomes.

## 2. Materials and Methods

### 2.1. Protocol and Research Question

The protocol for this systematic review conformed to the Preferred Reporting Items for Systematic Reviews and Meta-Analyses (PRISMA) statement and was registered in PROSPERO (registration number: CRD42020212603). The research questions “What oral antibiotics are prescribed as empirical therapy for acute dentoalveolar infections?”, “What is the regimen of oral antibiotics used for empirical therapy for acute dentoalveolar infections?” and “What are the clinical outcomes of these antibiotic regimens?” were investigated.

### 2.2. Search Strategies and Study Selection

In October 2020, three databases were searched from their earliest dates: Ovid Embase, Ovid Medline and Web of Science. The search strategies and terms were developed in consultation with an information specialist at the University of Melbourne and are shown in Supplementary Table S1. The search strategies included only human studies in English language, as resources were not available for translation. Original research studies from peer-reviewed journals included randomized controlled trials, comparative trials and prospective/retrospective studies assessing the use of oral antibiotics for empirical therapy to manage dentoalveolar infections. Studies involving parenteral antibiotics, studies where it was unclear if dentoalveolar infections were the primary cause of infection (e.g., head/neck space infections), studies where the antibiotic regimen or the route of administration of antibiotic was unclear, case studies, case reports, animal studies and reviews were excluded.

**Figure 1:**
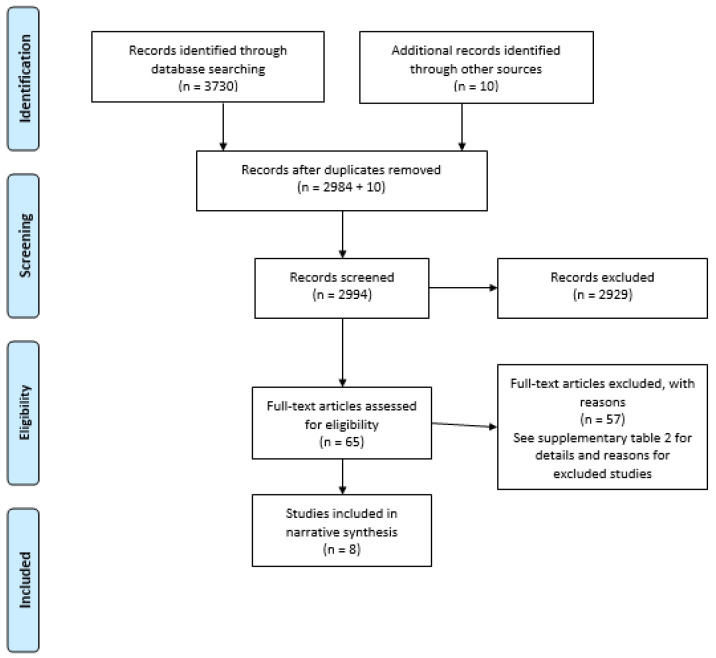
Preferred Reporting Items for Systematic Reviews and Meta-Analyses (PRISMA) Flow Diagram for study selection.

After performing the search, extracting all titles and abstracts into Endnote X9 and removing duplicates, two authors (LT and MC) screened all titles, abstracts and full texts independently for possible inclusion. Reference lists in review articles were also hand-searched for possible inclusion. Discrepancies were resolved with discussion after each round of screening. Figure 1 shows the summary of the selection process, and Supplementary Table S2 the reasons for the exclusion of articles.

### 2.3. Quality Assessment

The studies shortlisted for inclusion were quality-assessed using the validated 16-item Quality Assessment Tool for studies with Diverse Design (QATSDD) [27]. This was performed separately by LT and MC. Discrepancies were resolved through discussion. Studies were not included in the systematic review if they scored less than 50% in the QATSDD assessment.

### 2.4. Extraction of Antibiotics and Outcomes

Each article was assessed independently by LT and MC for the study protocol and design, objectives, clinical interventions, antibiotics and regimen used, the clinical outcomes measured for success and the results of the study with respect to the clinical outcomes. Outcomes that were not considered relevant to this study, such as microbiological analysis of samples, were excluded from the extraction process.

## 3. Results

### 3.1. Study Selection

After data extraction, 3740 articles were identified for possible inclusion into this review. After duplicates were removed, 2994 articles were screened, 65 articles were shortlisted for full-text review, and 57 were excluded with reasons. Nine articles met the inclusion criteria and underwent quality assessment, and eight were included in the study (Table S3).

### 3.2. Study Characteristics

The characteristics of the eight included studies are shown in Table 1 [4,24,28,29,30,31,32,33]. There were three prospective, double-blinded, randomised clinical studies, two prospective, randomised studies, two prospective studies and one retrospective study. All the participants had orofacial infections of dentoalveolar origin, including periapical, periodontal or pericoronal abscesses. One study included antibiotic prescribing for both dentoalveolar infections and gingival infiltrates, and the prescription for the latter was not included [24]. The studied population consisted mostly of adults (age > 16 years), although one study included paediatric patients [32]. The studies were published from 1983 to 2018. All studies included some participants that exhibited acute odontogenic infections with systemic signs, such as primary maxillofacial space swelling, temperature elevation and lymphadenopathy. 

The objective of all the studies was to evaluate the use of oral antibiotics for infections of dentoalveolar origin. All studies involved surgical or dental intervention to address the cause of the infection by extraction or incision/drainage through the root canal system or soft tissue, in addition to the prescription of antibiotics. All studies included cohorts that were administered antibiotics, except for two studies, i.e., Kumari et al. [32] and Matijevic et al. [31], which both presented a cohort of patients who received surgical intervention only.

### 3.3. Antibiotic Regimens Used

The antibiotics used in the studies included amoxicillin, amoxicillin with clavulanic acid, cefalexin, clindamycin, erythromycin, metronidazole, moxifloxacin, ornidazole and phenoxymethylpenicillin. Some of these were used as monotherapy or in combination, and the regimens of these antibiotics varied in dose and frequency. A penicillin antibiotic was trialled in all studies except one [24]. The spectrum of the antibiotics employed ranged from narrow (e.g., phenoxymethylpenicillin alone) to broad (amoxicillin with clavulanic acid). Metronidazole was used in combination with a penicillin, [4,32] or as monotherapy in one study [4]. Clindamycin was used as monotherapy in four studies [24,28,29,30]. The bacteria targeted would therefore have differed, with metronidazole being active against obligate anaerobes only, while penicillin antibiotics generally target Gram-positive microorganisms, facultative anaerobes and some obligate anaerobes. The treatment duration ranged from 2 to 7 days, with one study [4] indicating that patients received antibiotics until improvement, which was noted 2–3 days after the beginning of treatment. One study did not specify the treatment duration. The various antibiotic regimens are shown in Table 2.

### 3.4. Outcomes of Oral Antibiotics for Dentoalveolar Infections

All studies demonstrated clinical success with the use of the varying regimens of oral antibiotics and surgical intervention. Clinical improvement was noted in two studies after 2–3 days [4,30], in one study after 5 days [29], in two studies after 5–7 days [24,31], and in three studies after 7 days [28,32,33]. Kumari et al. [32] demonstrated that the cohort of patients who received incision and drainage only without oral antibiotics did not present any statistically significant differences in the examined parameters with respect to the group that received surgical intervention with a broad-spectrum combination antibiotic (amoxicillin with clavulanic acid and metronidazole). However, Matijevic et al. [31] showed that the clinical signs and symptoms of the group of patients who received amoxicillin or cephalexin together with drainage of the infection lasted 4.47 ± 0.62 and 4.67 ± 0.65 days, respectively, whereas the clinical signs and symptoms of the group of patients receiving surgical intervention only lasted 6.17 ± 0.81 days, (*p* < 0.05). Except for one study, none of the patients deteriorated, and for the majority of those who did not improve at the review visits, this was attributable to the inability to establish drainage at the initial appointment [30].

## 4. Discussion

A comprehensive evaluation of oral antibiotic regimens as adjunctive measures for the management of acute dentoalveolar infections and their corresponding clinical outcomes was undertaken. There was a range of antibiotics trialled, with varying spectrums and regimens used, and all produced similar clinical success. Interestingly, one study employed a broad-spectrum combination of amoxicillin with clavulanic acid in addition to metronidazole in one patient cohort versus no antibiotic in another group, and both arms of this study produced the same clinical outcomes [32]. Given the anaerobic coverage of amoxicillin with clavulanic acid, the additional benefit of metronidazole is unclear, but this review highlights the effectiveness of dental treatment to address the source of infection as being the primary factor in the successful management of dentoalveolar abscesses. It is unclear which antibiotic or regimen is the most effective to manage odontogenic infections in clinical practice, but the evidence here suggests that broad-spectrum antibiotics as first-line empirical therapy for infections with non-severe features, such as single-space extraoral swelling, are unnecessary since the narrow-spectrum antibiotic phenoxymethylpenicillin was also effective [4,28,29].

The antibiotics used in the studies varied from narrow- to broad-spectrum combinations, but all showed similar overall clinical outcomes of success. However, the included studies used a wide variety of clinical outcome measures ranging from patient-reported pain scores [24] to complete resolution of swelling or temperature [30], general treatment response [29], scored reduction [4] or complete resolution of overall clinical symptoms [24,31], objective clinical measurements of mouth opening [24,32] and resumption of normal life activities [32]. Adverse effects were described in some of the studies, to varying degrees [24,28,29]. The heterogeneity of antibiotics used and outcome parameters measured therefore precluded direct comparisons and undertaking a meta-analysis of these results. 

The relative importance of antibiotic treatment in the management of dentoalveolar infections remains unclear. One study [32] from this review that evaluated patients receiving a broad-spectrum combination of amoxicillin and clavulanic acid with metronidazole showed no difference in the clinical parameters of pain, mouth opening, swelling and purulent discharge, compared to the patients who did not receive any antibiotics [32]. However, another randomised study [31] showed that the use of antibiotics reduced the duration of signs and symptoms by approximately two days. Since there was no deterioration of patients who did not receive antibiotics in either study, further research would need to be undertaken to establish if antibiotics are in fact necessary in the management of non-severe primary space odontogenic infections, provided that drainage can be established in an otherwise healthy individual. An audit of antimicrobial prescribing for acute dentoalveolar infections further demonstrated that surgical drainage and removal of the cause of infection can manage the infection successfully without antibiotics [34]. Since Guralnick and Williams pioneered surgical drainage of anatomic spaces and the securing of a patent airway in the management of Ludwig’s angina in the 1940s, mortality has decreased significantly [35]. This review highlights the importance of addressing the cause of infection and establishing drainage, as most patients who did not improve were due to inadequate drainage [30]. In only one study, patients (n = 5) did not respond to antibiotic therapy, where phenoxymethylpenicillin was used [33]. The authors thought this could be due to the presence of penicillin-resistant *Bacteroides* species, as well as to the more favourable pharmacokinetics of ornidazole compared to phenoxymethylpenicillin [33]. However, details of the clinical situations were not provided, including the individual patients’ immune status, if any of these patients received dental treatment, as two patients in each cohort received antibiotics only, or if drainage was able to be achieved on the initial visit [33]. In addition, while this study was conducted in Sweden almost four decades ago [33], a recent longitudinal study on dental prescribing in Sweden and Norway shows that phenoxymethylpenicillin is currently most commonly prescribed [36]. The current clinical implications of this study are therefore questionable.

There does not appear to be any significant differences in the overall clinical outcomes with any of the antibiotics prescribed, but this review does suggest that clinical improvement can be achieved after short courses of antibiotics (2–5 days), as shown in several of the studies included in this review [4,24,29,30,31] and in another prospective audit [34]. It does also appear from these studies [4,32], as well as from other reviews, that tooth extraction or drainage through the soft tissues appears to lead to a faster resolution of infections compared to drainage through the root canal system [32,37,38,39].

The choice of antibiotics according to dental guidelines for the management of odontogenic infections has been based on antimicrobial susceptibility and resistance studies that have cultured bacteria from pus samples and formulated antibiotic recommendations depending on resistance rates [40]. Indeed, the Australian dental therapeutic guidelines are based on international susceptibility data from isolates obtained in Russia, Romania and Europe [41,42,43,44]. Interestingly, one study in this review demonstrated high levels of penicillin resistance (43%) in isolates cultured from samples of odontogenic infections in patients who received monotherapy with either phenoxymethylpenicillin or amoxicillin [4]. However, all patients had improved signs and symptoms on review 2–3 days later, and there was no significant difference in patients’ improvement scores in patients who had the presence of absence of penicillin-resistant bacteria [4]. Since the presence of penicillin resistance did not affect the outcome of treatment with penicillin, the authors of that study questioned the need for any type of antibiotic if adequate drainage can be achieved [4]. When drainage is not possible, for example because of diffuse cellulitis or trismus, antibiotic therapy likely has a more critical role, and the impact of resistance is greater in these situations [4]. This situation may be encountered in the outpatient clinical environment, where surgical intervention may be subsequently performed. In deep-space infections which require patient hospitalisation, penicillin resistance correlates with poorer clinical outcomes [22]. However, for a non-severe extra-oral swelling involving a primary maxillofacial space, the effects of bacterial resistance and the subsequent benefit of antibiotics are questioned, provided drainage can be established in an otherwise healthy person.

The most common bacteria isolated from odontogenic infections in these studies included oral species of *Prevotella, Peptostreptococcus*, *Streptococcus, Fusobacterium* and *Enterococcus faecalis,* amongst others, all demonstrating varying levels of resistance to the antibiotics tested [4,24,29,31,32]. One of the difficulties in determining the bacteria resistance profiles and the pathogenic isolates in dentoalveolar infections is represented by the complex interactions and relationships among the members of the oral microbiota in polymicrobial endodontic infections that can lead to additive or synergistic pathogenic effects and even the death of the resistant strain on surgical drainage [40]. This is reflected in the high levels of penicillin resistance exhibited in the study of Kuriyama and colleagues [4], while many of these patients responded to penicillin treatment. The oral cavity is colonised by a large and diverse range of organisms, of which, around 10% are regularly isolated using conventional techniques [45]. There are limitations inherent in the cultivation methods traditionally used to sample and grow suspected pathogenic microorganisms as well as related to the presence of bacterial strains that have not yet been cultivated [40]. One of the major limitations is time, as it usually takes 2–3 days to reliably determine the species present.. The identification of bacteria responsible for endodontic infections using culture-independent molecular genetic methods, such as polymerase chain reaction-based assays, has shown a much greater bacterial diversity than that previously identified using culturing techniques [46]. These methods also produce results rapidly, which is practical in the clinical setting. Even when bacterial species are commonly identified in samples taken from areas of infection, their presence may not necessarily be related to the cause of disease, as in the case of *E. faecalis* in post-endodontic treatment disease [46]. Additionally, by growing specific bacteria from a pus sample, the virulence of the isolates can be affected by their segregation in the culture medium, as well as by the lack of interaction with the remaining organisms that were present in the dentoalveolar infection in vivo [40].

As resistance profiles are likely to be more important clinically for dentoalveolar infections where drainage cannot be established [4], further research into the accurate profiling of these infections is worthwhile. Identifying the bacterial composition of the microbiota and their antibiotic susceptibilities in patients with dentoalveolar infections by assessing resident oral microbiota for the presence, occurrence and diversity of antimicrobial resistance genes and comparing this with data from matched controls is recommended. Future research could involve a microbiomic approach for the identification of bacteria, followed by a specific targeted method for the determination of antibiotic resistance genes. Only a subset of the oral microbiome will be found in odontogenic infections, and the determination of the species and resistome in a large number of these samples would be ideal. It is possible that when these species proliferate in localised infections their resistance profiles change, thus the determination of the actual species present and the (combinations of) resistance genes in an infection would be useful.

Publication bias and exclusion of studies other than English are possible limitations for this review. Some reported outcomes were subjective, such as patient-reported pain and “return to normal life”, so are at risk of reporting bias [24,32]. The trial designs of most studies were not blinded, and only two studies had a control arm with no antibiotics [31,32]. For ethical reasons, it can be argued that antibiotics would have to be prescribed due to the potential deterioration of odontogenic-space infections. However, the results of this present review suggest that future research should include a control arm without antibiotics, provided that drainage can be achieved, to investigate the necessity of prescribing antibiotics for such situations. People who have recently taken penicillin have had higher levels of penicillin-resistant bacteria isolated more frequently, and individuals who have been prescribed a course of antibiotics in primary care can develop bacterial resistance detectable up to 12 months later [47]. Further research can be directed towards assessing the most appropriate antibiotic on the basis of clinical outcomes, to determine the narrowest but effective spectrum of the antibiotic to be prescribed.

## 5. Conclusions

This review highlights the effectiveness of dental treatment to address the source of infection as the primary factor in the successful management of dentoalveolar abscesses. The ideal choice, regimen and spectrum of antibiotics as adjunctive management of localised dentoalveolar infections or involving a primary space, are unclear. In line with principles of antibiotic stewardship and the worldwide movement to narrow the spectrum of antibiotics used to prevent antibiotic resistance, it would appear from this review, that employing a broad-spectrum combination as first-line, empirical therapy for localised or primary space odontogenic infections is unnecessary where drainage has been established. Narrow-spectrum agents appear to be as effective in an otherwise healthy individual. Further research is required on the benefits of antibiotics in the management of non-severe extra-oral swellings, if drainage can be achieved. The antibiotic resistance of oral isolates and the concurrent resistome profiles of patients’ oral microbiome to more accurately guide antibiotic recommendations for odontogenic infections, especially where drainage cannot be established, should also be explored.

## Figures and Tables

**Table 1:** Characteristics of the included studies.

Study	Study Design	Objectives	Participants	Clinical Intervention	Definition of Clinical Outcomes	Outcomes
Von Konow and Nord, 1983 [33]	Prospective, randomised, double-blind study	To compare the efficacy of ornidazole to that of PMV in the treatment of orofacial infections	60 adult patients with acute orofacial infections. Ornidazole group: 14 males, 16 females, age range: 22–77 years Phenoxymethylpenicillin group: 17 males, 13 females, age range: 22–77 years	Surgical drainage was provided to all patients, except for two in each group, who received antimicrobial therapy only	Response to treatment was considered moderate or poor when the signs and symptoms of fever, swelling, pain and disturbance of sleep had not resolved or markedly subsided within 5 days	Ornidazole group: all patients cured in 7 days PMV group: 25 patients were cured in 7 days; 5 patients did not respond. Adverse effects: Ornidazole group: 1 patient reported metallic taste, 1 had feebleness, 1 had headache, and others had headache, weakness, nausea. Phenoxymethylpenicillin group: 3 patients had nausea
Gilmore et al., 1988 [28]	Prospective, randomised, double-blind clinical study	To compare the efficacy of PMV versus clindamycin in the treatment of moderate–severe orofacial infections of odontogenic origin	55 adult patients (41 males and 14 females) with moderate–severe orofacial infection of odontogenic origin	Incision and drainage procedure via an intraoral approach	Patients were seen at baseline and days 3 and 7. Success was defined as elimination of the infection in 7 days Improvement: decrease in signs/symptoms by day 7 but requiring an additional course of antibiotic Failure: increasing signs/symptoms of infection by day 7	PMV group: 22 patients (81%) had a successful outcome; 5 (19%) showed improvement Clindamycin group: 23 (82%) had a successful outcome; 5 (18%) showed improvement Adverse effects: PMV group: 1 patient had diarrhoea Clindamycin: 2 patients had diarrhoea (in 1 case, it was *C. difficile*-associated diarrhoea)
Von Konow et al., 1992 [29]	Prospective, randomised study	To compare penicillin and clindamycin with respect to microbiological and clinical efficacy and adverse effects in the treatment of orofacial infections	60 patients (36 males and 24 females, mean age 46 years, range 20–70 years), with acute dentoalveolar infections	Incision and drainage (where indicated)	Patients were examined on days 1, 3, 7 and 14. Treatment was regarded as poor when clinical symptoms had not disappeared or markedly subsided within 5 days, or when symptoms recurred during the observation period	Clinical outcomes: All patients except for one in each group responded to treatment Adverse effects: PMV group: 1 patient had severe diarrhoea Clindamycin group: 6 patients with moderate–severe gastrointestinal discomfort and 1 case *of C. difficile*-associated diarrhoea
Martin et al., 1997 [30]	Prospective clinical study over 3 years	To evaluate shortened courses of antibiotics in the management of dentoalveolar abscesses	759 patients (483 males and 276 females, age range: 16–81 years) with acute dentoalveolar abscesses associated with systemic signs (swelling, temperature elevation)	Drainage of the abscess by incision (124 patients) or extraction (635 patients)	Primary outcome: resolution of the swelling and a normal temperature	At 2–3 days, the primary outcome was achieved in: Amoxicillin group: 537/546 patients Clindamycin group: 140/141 patients Erythromycin group: 71/72 patients At 2–3 days, 748 patients had achieved the primary outcome and discontinued antibiotic therapy; 11 patients required re-incision of the abscess after 2–3 days
Kuriyama et al., 2005 [4]	Retrospective study	To determine if the outcome of treatment of dentoalveolar infection was influenced by the choice of antibiotic and the presence of penicillin-resistant bacteria.	112 patients (88 males and 24 females, age range: 17–81 years) with acute dentoalveolar infection	Surgical drainage through incision of the soft tissue swelling or through the pulp chamber	Clinical signs and symptoms were reassessed at 48 or 72 h. A four-point scale was used to measure success as follows: 3, Completely improved (complete resolution) 2, Much improved (almost complete resolution) 1, Slightly improved (the intensity of signs/symptoms slightly reduced)0, No improvement (same signs/symptoms as at baseline)	All antibiotic regimens produced a satisfactory outcome at 48 or 72 h, (mean score 2.3–2.6), with no significant differences in the regimens. Of the patients who underwent incisional drainage, the mean improvement score was 2.5
Matijevic et al., 2009 [31]	Prospective comparative study	To investigate the clinical efficiency of amoxicillin and cefalexin in the empirical treatment of acute odontogenic abscesses and assess the antimicrobial susceptibility of the isolated bacteria in early phases of its development	90 patients with acute odontogenic abscesses who received surgical treatment	Extraction of the tooth and/or abscess incision	Inflammatory swelling, regional lymphadenopathy, trismus, temperature were considered clinical symptoms of infection. Antibiotic therapy was stopped after full regression of all clinical symptoms	Amoxicillin group: 93.3% of patients had full recovery on the 5th day; signs and symptoms lasted for 4.47 days on average, but significant regression of swelling was recorded on the 2nd day for 22/30 patients. Cefalexin group: 90.0% of patients had full recovery on the 5th day; signs and symptoms lasted for 4.67 days on average; significant regression of swelling was recorded on the 2nd day for 23/30 patients. Surgical group: 93.3% of patients had full recovery on the 7th day; signs and symptoms lasted on average for 6.17 days, with significant regression of swelling on the 3rd day for 25/30 patients
Cachovan et al., 2011 [24]	Phase II, prospective, double-blind, randomised trial	To compare the efficacies and safeties of moxifloxacin and clindamycin for the treatment of patients with gingival inflammatory infiltrates and as adjuvant therapy for patients with odontogenic abscesses requiring surgical treatment.	31 patients (minimum age 18 years) with a diagnosis of odontogenic abscess (dentoalveolar, periodontal, pericoronitis) requiring surgical intervention and adjunctive antibiotic treatment	Surgical interventions in accordance with the guidelines of the German Society for Oral and Maxillofacial surgery, including surgical incisions, drainages, tooth extraction, debridement and puncture.	Pain reduction using a visual analogue scale at days 2–3 from baseline. Rating of cure: resolution of all signs of inflammation including fever, negative palpation for lymphadenopathy, subjectively unobstructed opening of the mouth and incisal edge distance of at least 35 mm, no need for further therapy. Improvement: signs of inflammation were decreased by at least 50%, body temperature ≤ 38.0 °C, reduced excretion of pus, soft/palpable lymph nodes, opening of the mouth was slightly obstructed, incisal edge distance was 35 mm or lower Failure: initial fever did not decrease, excretion of pus was unchanged, palpation for lymphadenopathy was positive	Pain reduction: Mean pain reduction on days 2–3 was higher for moxifloxacin compared to clindamycin, but the difference did not reach statistical significance. All patients had clinical outcomes rated as improved or cure in both the moxifloxacin group and the clindamycin group by days 5–7. The differences between the treatment groups did not reach statistical significance. Adverse effects: The rate was higher for clindamycin compared to moxifloxacin, especially nausea and diarrhoea
Kumari et al., 2018 [32]	Prospective, randomised clinical study	To compare treatment outcome of removal of foci and incision and drainage, with or without antibiotic therapy, in the management of single primary maxillofacial space infection with a known focus	40 patients (age range 10–50 years, mean: 27.3 years) with a single primary odontogenic maxillofacial space infection	Extraction or endodontics; surgical drainage (either extraoral or intraoral)	Patients were evaluated on days 1, 2, 3, 5 and 7. Pain, mouth opening, swelling, purulent discharge, return to normal life	Pain: The majority of patients in both groups were pain-free by day 7. The difference in the mean pain scores between groups A and B were clinically significant at any visit. Mouth opening: the percentage increase in mouth opening was 25% for the antibiotic group and 21% for the group without antibiotics between days 1 and days 7. Purulent discharge stopped within 3 days for 75% of the patients. Return to normal life: 47.5% of the patients reported a return to normal life on day 7. No significant differences between both groups for any category of clinical outcome

PMV: Phenoxymethylpenicillin.

**Table 2:** Antibiotic regimens.

Study	Number of Patients	Drug		Dose	Frequency	Duration
Von Konow and Nord, 1983 [33]	30	Ornidazole		500 mg	12-hourly	7 days
	30	Phenoxymethylpenicillin		800 mg	12-hourly	7 days
Gilmore et al., 1988 [28]	28	Clindamycin		150 mg	4/day	7 days
	27	Phenoxymethylpenicillin		250 mg	4/day	7 days
Von Konow et al., 1992 [29]	30	Clindamycin		150 mg	6-hourly	7 days
	30	Phenoxymethylpenicillin		1 g	12-hourly	7 days
Martin et al., 1997 [30]	546	Amoxicillin		250 mg	8-hourly	537/546 patients: 2–3 days; 9/546 patients: 10 days
	141	Clindamycin		150 mg	6-hourly	140/141 patients: 2–3 days; 1/141 patients: 10 days
	72	Erythromycin		250 mg	6-hourly	71/72 patients: 2–3 days; 1/72 patients: 10 days
Kuriyama et al., 2005 [4]	65		Amoxicillin	500 mg	8-hourly	2–3 days
			Phenoxymethylpenicillin	500 mg	6-hourly	
	24	Phenoxymethylpenicillin/Metronidazole		500 mg/400 mg	8-hourly/8-hourly	
	9	Metronidazole 400 mg		400 mg	8-hourly	
	6	Amoxicillin/Clavulanic Acid		375 mg (CA)	8-hourly	
	6	Erythromycin/Metronidazole		250 mg/400 mg	8-hourly/8-hourly	
	2	Erythromycin		250 mg	6-hourly	
Matijevic et al., 2009 [31]	30	Amoxicillin		500 mg	6-hourly	Until symptoms had resolved; 5 days
	30	Cefalexin		500 mg	6-hourly	
Cachovan et al., 2011 [24]	16	Clindamycin		300 mg	4/day	5 days
	15	Moxifloxacin		400 mg	1/day	5 days
Kumari et al., 2018 [32]	20	Amoxicillin/Clavulanic Acid and Metronidazole		625 mg and 400 mg	3/day	Unreported

## Data Availability

The data available in this study are presented in this article or the supplementary materials.

## Supplementary Materials

The following are available online at https://www.mdpi.com/2079-6382/10/3/240/s1, Table S1: Example of the search strategy used to identify relevant papers for this systematic review, Table S2: Excluded full-text articles with reasons for exclusion, Table S3: Methodological quality assessment of studies included in this systematic review across primary dental care using the Quality Assessment Tool for Studies with Diverse Design (QATSSD).
