# Supplementary material for: Oral Antibiotic for Empirical Management of Acute Dentoalveolar Infections—A Systematic Review
Source: Antibiotics (Basel). 2021 Feb 28;10(3):240. doi: 10.3390/antibiotics10030240 (PMC7997333; doi:10.3390/antibiotics10030240)
Supplement: Supplementary file 1 [file antibiotics-10-00240-s001.pdf]

Supplementary Material

S1. Example of the search strategy used to identify relevant papers for the systematic review.

1. “Drug Resistance, Microbial” mp. [mp = title, abstract, original title, name of substance word, subject heading word, floating sub-heading word, keyword heading word, organism supplementary concept word, protocol supplementary concept word, rare disease supplementary concept word, unique identifier, synonyms]

2. antibiotic mp. [mp = title, abstract, original title, name of substance word, subject heading word, floating sub-heading word, keyword heading word, organism supplementary concept word, protocol supplementary concept word, rare disease supplementary concept word, unique identifier, synonyms]

3. antimicrobial mp. [mp = title, abstract, original title, name of substance word, subject heading word, floating sub-heading word, keyword heading word, organism supplementary concept word, protocol supplementary concept word, rare disease supplementary concept word, unique identifier, synonyms]

4. “Infection Control, Dental” mp. [mp = title, abstract, original title, name of substance word, subject heading word, floating sub-heading word, keyword heading word, organism supplementary concept word, protocol supplementary concept word, rare disease supplementary concept word, unique identifier, synonyms]

5. 1 or 2 or 3 or 4

6. tooth mp. [mp = title, abstract, original title, name of substance word, subject heading word, floating sub-heading word, keyword heading word, organism supplementary concept word, protocol supplementary concept word, rare disease supplementary concept word, unique identifier, synonyms]
7. odontogenic mp. [mp = title, abstract, original title, name of substance word, subject heading word, floating sub-heading word, keyword heading word, organism supplementary concept word, protocol supplementary concept word, rare disease supplementary concept word, unique identifier, synonyms]
8. “periapical” mp. [mp = title, abstract, original title, name of substance word, subject heading word, floating sub-heading word, keyword heading word, organism supplementary concept word, protocol supplementary concept word, rare disease supplementary concept word, unique identifier, synonyms]
9. pericoron*mp. [mp = title, abstract, original title, name of substance word, subject heading word, floating sub-heading word, keyword heading word, organism supplementary concept word, protocol supplementary concept word, rare disease supplementary concept word, unique identifier, synonyms]
10. periodont*mp. [mp = title, abstract, original title, name of substance word, subject heading word, floating sub-heading word, keyword heading word, organism supplementary concept word, protocol supplementary concept word, rare disease supplementary concept word, unique identifier, synonyms]
11. endodontic mp. [mp = title, abstract, original title, name of substance word, subject heading word, floating sub-heading word, keyword heading word, organism supplementary concept word, protocol supplementary concept word, rare disease supplementary concept word, unique identifier, synonyms]
12. head mp. [mp = title, abstract, original title, name of substance word, subject heading word, floating sub-heading word, keyword heading word, organism supplementary concept word, protocol supplementary concept word, rare disease supplementary concept word, unique identifier, synonyms]

13. neck mp. [mp = title, abstract, original title, name of substance word, subject heading word, floating sub-heading word, keyword heading word, organism supplementary concept word, protocol supplementary concept word, rare disease supplementary concept word, unique identifier, synonyms]
14. “root canal” mp. [mp = title, abstract, original title, name of substance word, subject heading word, floating sub-heading word, keyword heading word, organism supplementary concept word, protocol supplementary concept word, rare disease supplementary concept word, unique identifier, synonyms]

15. 6 or 7 or 8 or 9 or 10 or 11 or 12 or 13 or 14

16. infection* mp. [mp = title, abstract, original title, name of substance word, subject heading word, floating sub-heading word, keyword heading word, organism supplementary concept word, protocol supplementary concept word, rare disease supplementary concept word, unique identifier, synonyms]
17. abscess* mp. [mp = title, abstract, original title, name of substance word, subject heading word, floating sub-heading word, keyword heading word, organism supplementary concept word, protocol supplementary concept word, rare disease supplementary concept word, unique identifier, synonyms]
18. swelling mp. [mp = title, abstract, original title, name of substance word, subject heading word, floating sub-heading word, keyword heading word, organism supplementary concept word, protocol supplementary concept word, rare disease supplementary concept word, unique identifier, synonyms]
19. 16 or 17 or 18
20. “clinical trial” mp. [mp = title, abstract, original title, name of substance word, subject heading word, floating sub-heading word, keyword heading word, organism supplementary concept word, protocol supplementary concept word, rare disease supplementary concept word, unique identifier, synonyms]
21. cohort mp. [mp = title, abstract, original title, name of substance word, subject heading word, floating sub-heading word, keyword heading word, organism supplementary concept word, protocol supplementary concept word, rare disease supplementary concept word, unique identifier, synonyms]
22. “case control” mp. [mp = title, abstract, original title, name of substance word, subject heading word, floating sub-heading word, keyword heading word, organism supplementary concept word, protocol supplementary concept word, rare disease supplementary concept word, unique identifier, synonyms]
23. prospective mp. [mp = title, abstract, original title, name of substance word, subject heading word, floating sub-heading word, keyword heading word, organism supplementary concept word, protocol supplementary concept word, rare disease supplementary concept word, unique identifier, synonyms]
24. retrospective mp. [mp = title, abstract, original title, name of substance word, subject heading word, floating sub-heading word, keyword heading word, organism supplementary concept word, protocol supplementary concept word, rare disease supplementary concept word, unique identifier, synonyms]
25. 20 or 21 or 22 or 23 or 24

26. 5 and 15 and 19 and 25

Table S2 Excluded full-text articles with reasons for exclusion.

ARTICLE	REASON CODE
Adamson OO, Gbotolorun OM, Odeniyi O, Oduyebo OO, Adeyemo WL. Assessment of predictors of treatment outcome among patients with bacterial odontogenic infection. Saudi Dental Journal. 2018;30(4):337-41.	1,6
Al-Malik M, Al-Sarheed M. Pattern of management of oro-facial infection in children: A retrospective. Saudi Journal of Biological Sciences. 2017;24(6):1375-9.	2,4
Almeida M, Tavares RS, Mourao J, Carvalho JFC. Deep neck infections with dental origin - 105 cases review. [Portuguese, English]. Revista Portuguesa de Estomatologia, Medicina Dentaria e Cirurgia Maxilofacial. 2013;54(4):197-202.	3
Al-Qamachi LH, Aga H, McMahon J, Leanord A, Hammersley N. Microbiology of odontogenic infections in deep neck spaces: a retrospective study. British Journal of Oral & Maxillofacial Surgery. 2010;48(1):37-9.	2
Altug HA, Erdogan O, Sahin S, Sencimen M, Okcu KM. Diagnosis and management of orofacial infections: A retrospective study of 440 patients. [Turkish]. Turkiye Klinikleri Journal of Medical Sciences. 2010;30(3):1002-8.	3
Bahl R, Sandhu S, Singh K, Sahai N, Gupta M. Odontogenic infections: Microbiology and management. Contemporary Clinical Dentistry. 2014;5(3):307-11.	2
Bakathir AA, Moos KF, Ayoub AF, Bagg J. Factors Contributing to the Spread of Odontogenic Infections: A prospective pilot study. Sultan Qaboos University Medical Journal. 2009;9(3):296-304.	2
Bali R, Sharma P, Gaba S. Use of metronidazole as part of an empirical antibiotic regimen after incision and drainage of infections of the odontogenic spaces. British Journal of Oral & Maxillofacial Surgery. 2015;53(1):18-22.	2
Baum SH, Ha-Phuoc AK, Mohr C. Treatment of odontogenic abscesses: comparison of primary and secondary removal of the odontogenic focus and antibiotic therapy. Oral Maxillofac Surg. 2020;24(2):163-72.	2
Bertossi D, Barone A, Iurlaro A, Marconcini S, De Santis D, Finotti M, et al. Odontogenic Orofacial Infections. Journal of Craniofacial Surgery. 2017;28(1):197-202.	4
Bottger S, Lautenbacher K, Domann E, Howaldt HP, Attia S, Streckbein P, et al. Indication for an additional postoperative antibiotic treatment after surgical incision of serious odontogenic abscesses. Journal of Cranio-Maxillo-Facial Surgery. 2020;48(3):229-34.	2
Bowe CM, O'Neill MA, O'Connell JE, Kearns GJ. The surgical management of severe dentofacial infections (DFI)-a prospective study. Irish Journal of Medical Science. 2019;188(1):327-31.	2
Bross-Soriano D, Arrieta-Gomez JR, Prado-Calleros H, Schimelmitz-Idi J, Jorba-Basave S. Management of Ludwig's angina with small neck incisions: 18 years experience. Otolaryngology - Head & Neck Surgery. 2004;130(6):712-7.	2
Cachovan G, Phark JH, Schon G, Pohlenz P, Platzer U. Odontogenic infections: an 8-year epidemiologic analysis in a dental emergency outpatient care unit. Acta Odontologica Scandinavica. 2013;71(3-4):518-24.	1
Chomarat M, Dubost J, Kalfon F. [Randomized study of amoxicillin-clavulanic acid versus amoxicillin-metronidazole combinations in pyogenic infections of dental origin]. Pathologie Biologie. 1991;39(5):558-60.	3
Daramola OO, Flanagan CE, Maisel RH, Odland RM. Diagnosis and treatment of deep neck space abscesses. Otolaryngology - Head and Neck Surgery. 2009;141(1):123-30.	4
Dodson TB, Perrott DH, Kaban LB. Pediatric maxillofacial infections: A retrospective study of 113 patients. Journal of Oral and Maxillofacial Surgery. 1989;47(4):327-30.	4
Dvori S, Laviv A, Rahima H, Taicher S. [Clinical parameters in evaluating hospitalized patients with orofacial odontogenic infection--a preliminary retrospective study]. Refuat Hapeh Vehashinayim. 2006;24(3):46-9, 93.	3
Ellison SJ. An outcome audit of three day antimicrobial prescribing for the acute dentoalveolar abscess. Br Dent J. 2011;211(12):591-4.	5
Gams K, Shewale J, Demian N, Khalil K, Banki F. Characteristics, length of stay, and hospital bills associated with severe odontogenic infections in Houston, TX. Journal of the American Dental Association. 2017;148(4):221-9.	4

Goncalves L, Lauriti L, Yamamoto MK, Luz JG. Characteristics and management of patients requiring hospitalization for treatment of odontogenic infections. Journal of Craniofacial Surgery. 2013;24(5):e458-62.	2,6
Gotz C, Reinhart E, Wolff KD, Kolk A. Oral soft tissue infections: causes, therapeutic approaches and microbiological spectrum with focus on antibiotic treatment. Journal of Cranio-Maxillofacial Surgery. 2015;43(9):1849-54.	2,6
Herrera D, Roldan S, O'Connor A, Sanz M. The periodontal abscess (II). Short-term clinical and microbiological efficacy of 2 systemic antibiotic regimes. Journal of Clinical Periodontology. 2000;27(6):395-404.	7
Kim MK, Chuang SK, August M. Antibiotic Resistance in Severe Orofacial Infections. Journal of Oral and Maxillofacial Surgery. 2017;75(5):962-8.	2
Larawin V, Naipao J, Dubey SP. Head and neck space infections. Otolaryngology - Head and Neck Surgery. 2006;135(6):889-93.	4
Hardjawinata K, Mangundjaja S, Sartono KR. Sensitivity of Bacterial Isolates in Odontogenic Abscesses and Clinical Response to Clindamycin. Asian Journal of Oral and Maxillofacial Surgery. 2003;15(4):250-5.	6
Lee YQ, Kanagalingam J. Deep neck abscesses: the Singapore experience. European Archives of Oto-Rhino-Laryngology. 2011;268(4):609-14.	4
Liau I, Han J, Bayetto K, May B, Goss A, Sambrook P, et al. Antibiotic resistance in severe odontogenic infections of the South Australian population: a 9-year retrospective audit. Australian Dental Journal. 2018;63(2):187-92.	4
Lin YT, Lu PW. Retrospective study of pediatric facial cellulitis of odontogenic origin. Pediatric Infectious Disease Journal. 2006;25(4):339-42.	4
Mahmoodi B, Weusmann J, Azaripour A, Braun B, Walter C, Willershausen B. Odontogenic Infections: A 1-year Retrospective Study. Journal of Contemporary Dental Practice [Electronic Resource]. 2015;16(4):253-8.	1,2
Nadig K, Tayloras NG. Management of odontogenic infection at a district general hospital. British Dental Journal. 2018;224(12):962-6.	2
Odzili FAI, Guimbi KM, Boumandoki PJC, Otiobanda GF, Ovoundard M, Ondzotto G. 67 cases of face and neck cellulitis managed at the Brazzaville Teaching Hospital. Revue De Stomatologie De Chirurgie Maxillo-Faciale Et De Chirurgie Orale. 2014;115(6):349-52.	3
Peters ES, Fong B, Wormuth DW, Sonis ST. Risk factors affecting hospital length of stay in patients with odontogenic maxillofacial infections. Journal of Oral & Maxillofacial Surgery. 1996;54(12):1386-91; discussion 91-2.	2
Plum AW, Mortelliti AJ, Walsh RE. Microbial flora and antibiotic resistance in odontogenic abscesses in Upstate New York. Ear, Nose, & Throat Journal. 2018;97(1-2):E27-E31.	1,4
Pourdanesh F, Dehghani N, Azarsina M, Malekhosein Z. Pattern of odontogenic infections at a tertiary hospital in tehran, iran: a 10-year retrospective study of 310 patients. Journal of Dentistry / Tehran University of Medical Sciences. 2013;10(4):319-28.	1,2
Rasteniene R, Puriene A, Aleksejuniene J, Peciuliene V, Zaleckas L. Odontogenic maxillofacial infections: A ten-year retrospective analysis. Surgical Infections. 2015;16(3):305-12.	2
Ritwik P, Fallahi S, Yu QZ. Management of facial cellulitis of odontogenic origin in a paediatric hospital. International Journal of Paediatric Dentistry. 2020;30(4):483-8.	2,4
Sanchez R, Mirada E, Arias J, Pano JR, Burgueno M. Severe odontogenic infections: Epidemiological, microbiological and therapeutic factors. Medicina Oral, Patologia Oral y Cirugia Bucal. 2011;16(5):e670-e6.	2
Sasaki J, Morishima T, Sakamoto H, Takai H, Ikeshima K, Shiiki K, et al. [Clinical evaluation of clarithromycin in treatment of acute dental infections. Comparative double-blind study using josamycin as the control]. Japanese Journal of Antibiotics. 1989;42(4):983-1013.	3
Sasaki J, Yamada Y, Morihana K, Kaneko A, Takai H, Ohmura H, et al. [Clinical evaluation of the TMS-19-Q.GC tablet in odontogenic infections. A comparative double-blind study with josamycin]. Japanese Journal of Antibiotics. 1985;38(5):1389-419.	3
Sasaki J, Yamada Y, Takai H. Clinical studies on TMS-19-Q.O tablets, the preparation of a new macrolide antibiotic, in the field of oral surgery. [Japanese]. Japanese Journal of Antibiotics. 1985;38(3):615-33.	3
Tomita S, Kasai S, Ihara Y, Imamura K, Kita D, Ota K, et al. Effects of systemic administration of sitafloxacin on subgingival microflora and antimicrobial susceptibility profile in acute periodontal lesions. Microbial Pathogenesis. 2014;71-72:1-7.	6
Ubirajara Sennes L, Imamura R, Veiga Angelico Jr F, Simoceli L, Frizzarini R, Hiroshi Tsuji D. Deep neck infections: Prospective study of 57 patients. Revista Brasileira de Otorrinolaringologia. 2002;68(3):388-93.	3
Uittamo J, Lofgren M, Hirvikangas R, Furuholm J, Snall J. Severe odontogenic infections: focus on more effective early treatment. British Journal of Oral and Maxillofacial Surgery. 2020;58(6):675-80.	4

Wang J, Ahani A, Pogrel MA. A five-year retrospective study of odontogenic maxillofacial infections in a large urban public hospital. International Journal of Oral & Maxillofacial Surgery. 2005;34(6):646-9.	2,6
Yuvaraj V. Maxillofacial Infections of Odontogenic Origin: Epidemiological, Microbiological and Therapeutic Factors in an Indian Population. Indian Journal of Otolaryngology & Head & Neck Surgery. 2016;68(4):396-9.	1
Zirk M, Buller J, Goeddertz P, Rothamel D, Dreiseidler T, Zoller JE, et al. Empiric systemic antibiotics for hospitalized patients with severe odontogenic infections. Journal of Cranio-Maxillo-Facial Surgery. 2016;44(8):1081-8.	2
Zirk M, Zoeller JE, Peters F, Ringendahl L, Buller J, Kreppel M. Cefazolin versus ampicillin/sulbactam as an empiric antibiosis in severe odontogenic neck infection descending from the lower jaw-retrospective analysis of 350 cases. Clinical Oral Investigations. 2020;10:10.	2
Tancawan AL, Pato MN, Abidin KZ, Asari AS, Thong TX, Kochhar P, et al. Amoxicillin/Clavulanic Acid for the Treatment of Odontogenic Infections: A Randomised Study Comparing Efficacy and Tolerability versus Clindamycin. Int J Dent. 2015;2015:472470.	7
Adriaenssen CF. Comparison of the efficacy, safety and tolerability of azithromycin and co-amoxiclav in the treatment of acute periapical abscesses. J Int Med Res. 1998;26(5):257-65.	7
Fazakerley MW, McGowan P, Hardy P, Martin MV. A comparative study of cephradine, amoxycillin and phenoxymethylpenicillin in the treatment of acute dentoalveolar infection. Br Dent J. 1993;174(10):359-63.	2
Lewis MA, Carmichael F, MacFarlane TW, Milligan SG. A randomised trial of co-amoxiclav (Augmentin) versus penicillin V in the treatment of acute dentoalveolar abscess. Br Dent J. 1993;175(5):169-74.	2
Al-Nawas B, Walter C, Morbach T, Seitner N, Siegel E, Maeurer M, et al. Clinical and microbiological efficacy of moxifloxacin versus amoxicillin/clavulanic acid in severe odontogenic abscesses: a pilot study. Eur J Clin Microbiol Infect Dis. 2009;28(1):75-82.	2
Davis WM, Jr., Balcom JH, 3rd. Lincomycin studies of drug absorption and efficacy. An evaluation by double-blind technique in treatment of odontogenic infections. Oral Surg Oral Med Oral Pathol. 1969;27(5):688-96.	2
Deffez JP, Scheimberg A, Rezvani Y. Multicenter double-blind study of the efficacy and tolerance of roxithromycin versus erythromycin ethylsuccinate in acute orodental infection in adults. Odontogenic Infections Study Group. Diagn Microbiol Infect Dis. 1992;15(4 Suppl):133S-7S.	2
Al-Belasy FA, Hairam AR. The efficacy of azithromycin in the treatment of acute infraorbital space infection. Journal of Oral & Maxillofacial Surgery. 2003;61(3):310-6.	4
Mangundjaja S, Hardjawinata K. Clindamycin versus ampicillin in the treatment of odontogenic infections. Clinical Therapeutics. 1990;12(3):242-9.	8

1 = no clinical outcomes detailed

2 = route of administration of antibiotic not specified, or parenteral antibiotics

3 = language other than English

4 = insufficient details of antibiotic regimen or insufficient details of treatment of odontogenic infection

5 = effectiveness of different antibiotics was not compared

6 = no comparison group

7 = localised dentoalveolar infections

8 = failed quality assessment (QATSDD)

Table S3. Methodological quality assessment of studies included in this systematic review using the Quality Assessment Tool for Studies with Diverse Design (QATSSD). The second mark relates to the scores of the second marker who undertook a parallel assessment of all studies in this systematic review.

	1. Explicit theoretical framework	2. Statement of aims/objectives in the main body of the report	3. Clear description of the research setting	4. Evidence of sample size considered in for the analysis	5. Representative sample of target group of a reasonable size	6. Description of the procedure for data collection	7. Rationale for the choice of data collection tool(s)	8. Detailed recruitment data	9. Statistical assessment of reliability and validity of the measurement tool(s)
Von Konow and Nord, 1983	3 and 3	3 and 3	3 and 3	0 and 0	1 and 1	3 and 3	1 and 2	2 and 1	0 and 0
Gilmore et al, 1988	3 and 3	3 and 3	1 and 2	0 and 0	1 and 1	3 and 3	2 and 2	1 and 1	0 and 0
Mangundjaja and Hardjawinata, 1990	3 and 3	3 and 3	1 and 0	0 and 0	2 and 1	3 and 3	1 and 2	1 and 0	0 and 0
Von Konow et al, 1992	3 and 3	3 and 3	3 and 3	0 and 0	1 and 1	3 and 3	2 and 2	1 and 1	0 and 0
Martin et al, 1997	3 and 3	3 and 3	3 and 3	0 and 0	1 and 2	3 and 3	2 and 3	2 and 3	0 and 0
Kuriyama et al, 2005	3 and 3	3 and 3	3 and 3	0 and 0	1 and 2	3 and 3	1 and 1	3 and 3	0 and 0
Matijevic et al, 2009	3 and 3	3 and 3	3 and 3	0 and 0	1 and 1	3 and 3	0 and 0	3 and 3	0 and 0
Cachovan et al, 2011	3 and 3	3 and 3	3 and 3	3 and 3	2 and 1	3 and 3	2 and 3	3 and 3	0 and 0
Kumari et al, 2018	2 and 3	3 and 3	3 and 3	3 and 3	2 and 1	3 and 3	0 and 0	3 and 3	0 and 0

Table S3 (continued)

	10. Fit between stated research question and method of data collection	11. Fit between stated research question and format and content of data collection tool, e.g., interview schedule	12. Fit between research question and method of analysis (N/A)	13. Good justification for the analytical method selected	14. Assessment of reliability of analytical process	15. Evidence of user involvement in the study design	16. Strengths and limitations critically discussed	Total	% Total	Average total
Von Konow and Nord, 1983	3 and 3	3 and 3	-	0 and 0	-	0 and 0	0 and 0	22 and 22	52 and 52	52
Gilmore et al, 1988	3 and 3	3 and 3	-	1 and 0	-	0 and 0	1 and 1	22 and 22	52 and 52	52
Mangundjaja and Hardjawinata, 1990	3 and 3	3 and 3	-	0 and 0	-	0 and 0	0 and 0	20 and 18	48 and 43	45
Von Konow et al, 1992	2 and 3	3 and 3	-	1 and 1	-	0 and 0	0 and 0	22 and 23	52 and 55	54
Martin et al, 1997	3 and 3	3 and 3	-	1 and 0	-	0 and 0	0 and 0	24 and 26	57 and 62	60
Kuriyama et al, 2005	3 and 3	3 and 2	-	1 and 1	-	0 and 0	2 and 2	26 and 26	62 and 62	62
Matijevic et al, 2009	3 and 3	3 and 3	-	1 and 0	-	0 and 0	0 and 0	23 and 22	55 and 52	54
Cachovan et al, 2011	3 and 3	3 and 3	-	2 and 3	-	1 and 0	3 and 3	35 and 33	83 and 79	81
Kumari et al, 2018	3 and 3	3 and 3	-	1 and 1	-	0 and 0	2 and 3	28 and 29	67 and 69	68
